# The Role of Lung Ultrasound in Low‐Resource Settings During the Coronavirus (SARS‐CoV‐2) Pandemic

**DOI:** 10.1002/jum.15755

**Published:** 2021-05-24

**Authors:** Giovanni Cappa, Gianmarco Secco, Andi Nganso, Ron Ruzga, Stefano Perlini

**Affiliations:** ^1^ Emergency Medicine Unit and Emergency Medicine Postgraduate Training Program, IRCCS Policlinico San Matteo University Hospital University of Pavia Pavia; ^2^ Italian Red Cross Rome Italy; ^3^ Medical Graduate University of Pavia Pavia Italy

**Keywords:** Covid‐19, evacuation, humanitarian, lung ultrasound, migration

## Abstract

Lung ultrasound (LUS) has proven to be a helpful diagnostic tool for evaluating lung involvement in respiratory pathologies. The usage of this imaging technique became even more widespread during the SARS‐CoV‐2 pandemic. The latest generation ultrasound scanners are conveniently portable and this permits ultrasound examinations to be performed even in extreme environments where no other diagnostic tool is available. Our team has developed the first guide that assists the clinician while operating in low‐resource settings, in managing a SARS‐CoV‐2 patient based on the clinical examination and the LUS findings.

Lung ultrasound (LUS) has become an important diagnostic tool in assessing respiratory pathologies, and several studies have confirmed its accuracy in determining the extension of lung involvement.^1^, ^2^

This imaging technique has gained an established role during the SARS‐CoV‐2 pandemic:^3^, ^4^ LUS scores may help the clinician in recognizing a diffused interstitial pneumonia and estimate the prognosis.^5^

In low resource settings, where a simple chest x‐ray is not available, LUS becomes an extremely beneficial diagnostic tool: the clinical examination is integrated with lung ultrasound and it profoundly aids in the subsequent clinical approach.

Operating in a scarce and limited supply scenario, the clinician must prioritize resources: the clinician decides which patient will benefit the most from treatment and when to evacuate a patient, if the option is available.

In the case of humanitarian medicine, there is also a great disproportion between the health care personnel and the patients; medical and the oxygen supply are often limited.

Medical evacuation of a patient is a procedure that is costly and requires a lot of resources: it must be considered as a final solution and it is not always available. In this article, we propose a flowchart to guide the clinician in this decision‐making process.

Our team took part onboard one of the Italian Red Cross quarantine ships, accommodating newly arriving North African migrants. Once they are salvaged, they must stay in isolation for their mandatory quarantine.

The Italian Red Cross medical team has the duty to provide first aid to whom that requires such, follow‐up SARS‐Cov‐2 patients onboard, and decide whether a patient needs an urgent medical evacuation. Neither laboratory test nor imaging diagnostic tool was available onboard.

Our staff brought onboard a handheld Clarius C3 ultrasound scanner (Clarius Mobile Health, 130–2985 Virtual Way, Vancouver, BC, V5M 4X7 Canada) that works with a rechargeable battery and transmits the image directly to a smartphone through a wireless connection.

We conducted constant follow‐up of SARS‐Cov‐2 patients, and we performed LUS on each individual who presented any respiratory symptom.

The lung involvement was quantified with the score we routinely use in our Emergency Department:^6^ we perform a 12‐pulmonary windows approach: it is faster than other proposed approaches, but nonetheless provides a comprehensive evaluation of the lung involvement. The ultrasound scanner probe is placed on the intercostal spaces and acoustic artifacts are examined: A‐lines suggest an aerated lung portion, whereas multiple B‐lines suggest a decreased aeration of the scanned lung segment^7^ (Figure 1).

**Figure 1:**
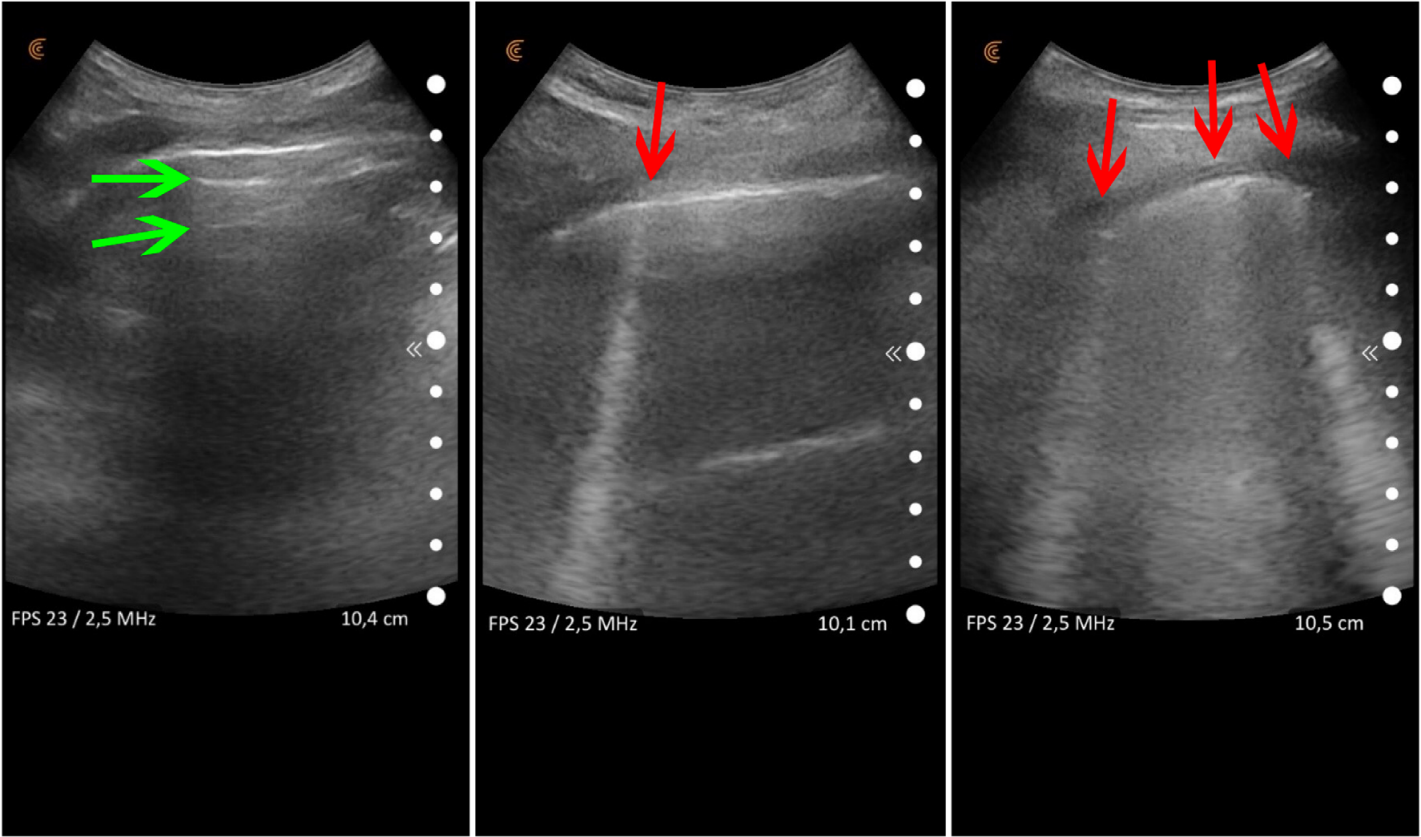
Acoustic artifacts in lung ultrasound: A‐lines (horizontal artifacts indicated by the green arrows) are the reverberation of the pleural line and suggest that the lung is aerated. B‐lines are vertical artifacts (indicated by the red arrows) that suggest a progressive decrease of lung aeration as the number of B‐lines increases.

We elaborated a flowchart, based on our experience, that guides the clinician in the decision‐making process (Figure 2).

**Figure 2:**
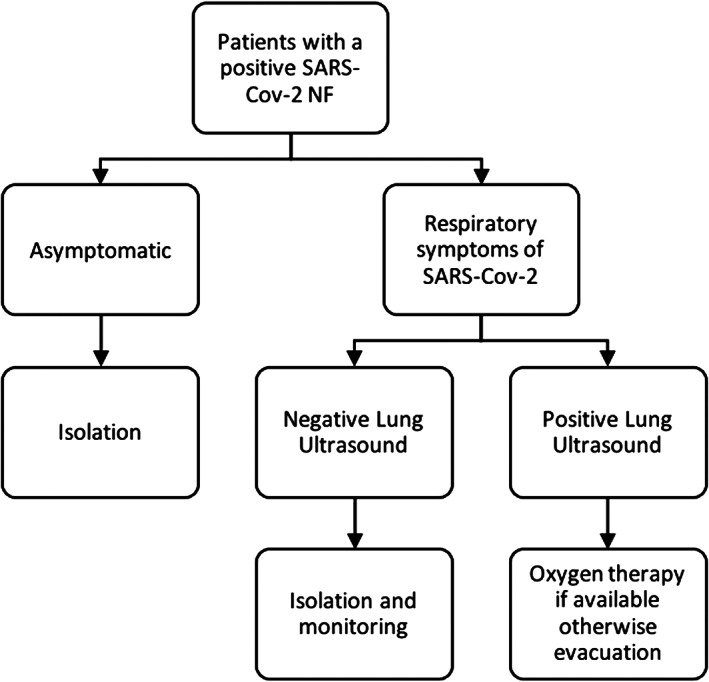
This flowchart guides the clinician in deciding when a patient should be evacuated, according to the patient's symptoms and LUS findings.

When should I plan a medical evacuation for a patient? This decision is based on the evidence that a high lung ultrasound score is predictive of a clinical worsening of the patient.

Several studies confirmed that the presence of a B pattern in multiple acoustic windows is related to a worsening of the clinical condition of a patient with Covid‐19. These patients are candidate for oxygen therapy and medical evacuation when the oxygen supply is limited or unavailable.

The role of ultrasound in the evaluation of the pulmonary involvement in patients with Covid‐19 pneumonia has been discussed in multiple studies but it gains even more importance if it is the only imaging technique available to the clinician.
